# The Use of NeuroAiD (MLC601) in Postischemic Stroke Patients

**DOI:** 10.1155/2012/506387

**Published:** 2012-12-13

**Authors:** Jose C. Navarro, Mark C. Molina, Alejandro C. Baroque II, Johnny K. Lokin

**Affiliations:** Stroke Unit, Department of Neurology and Psychiatry, University of Santo Tomas Hospital, España Boulevard, San Vicente Ferrer Ward, 1008 Manila, Philippines

## Abstract

*Aim*. We aimed to assess the efficacy of MLC601 on functional recovery in patients given MLC601 after an ischemic stroke. *Methods*. This is a retrospective cohort study comparing poststroke patients given open-label MLC601 (*n* = 30; 9 female) for three months and matching patients who did not receive MLC601 from our Stroke Data Bank. Outcome assessed was modified Rankin Scale (mRS) at three months and analyzed according to: (1) achieving a score of 0-2, (2) achieving a score of 0-1, and (3) mean change in scores from baseline. *Results*. At three months, 21 patients on MLC601 became independent as compared to 17 patients not on MLC601 (OR 1.79; 95% CI 0.62–5.2; *P* = 0.29). There were twice as many patients (*n* = 16) on MLC601 who attained mRS scores similar to their prestroke state than in the non-MLC601 group (*n* = 8) (OR 3.14; 95% CI 1.1–9.27; *P* = 0.038). Mean improvement in mRS from baseline was better in the MLC601 group than in the non-MLC601 group (−1.7 versus −0.9; mean difference −0.73; 95% CI −1.09 to −0.38; *P* < 0.001). *Conclusion*. MLC601 improves functional recovery at 3 months postischemic stroke. An ongoing large randomized control trial of MLC601 will help validate these results.

## 1. Introduction

There are currently few therapeutic options for acute ischemic strokes which are mainly limited to revascularization, antithrombotic agents and admission to a stroke unit [1–4]. Neuroprotection trials in acute ischemic stroke have consistently failed [5–7]. Furthermore, aside from rehabilitation, postacute stage long-term options for improving poststroke disabilities have not generated enough interest to be adequately addressed by pharmacological interventions. 

Recently, many studies have been published on the efficacy and safety of MLC601 (NeuroAiD) in improving functional and neurological outcomes among nonacute poststroke patients [8–15]. MLC601 has been registered in the Philippines since 2006. For several years now, we have had the opportunity to use MLC 601 in patients with ischemic stroke. Practitioners prescribe it to poststroke patients at a dose used in an ongoing large randomized controlled trial, that is, four capsules three times daily for 3 months [16]. 

It is the aim of this study to present our experience on the usefulness of MLC601 in ischemic stroke by assessing its efficacy on recovery from functional disability.

## 2. Methods

### 2.1. Study Design

This is a nested retrospective cohort study of patients in our Stroke Data Bank who were diagnosed with acute ischemic stroke confirmed by cranial computed tomography (CT) scan or magnetic resonance imaging (MRI). The Stroke Data Bank was duly approved by the institution for research and data analysis purposes and follows the Helsinki declaration on the rights of the patients. For this particular analysis, patients who received NeuroAiD (MLC601) during the course of their medical care from 2008 to 2011 were included and individually matched based on age and gender with an equal number of stroke patients who did not receive MLC601. 

### 2.2. Patients

In this analysis, patients were identified and included in the MLC601 group if they were 18 years old or older, had a prestroke modified Rankin Scale (mRS) score of less than or equal to 1, presented with cerebral infarction with compatible cranial CT scan or MRI findings, started on MLC601 within 6 months of stroke onset and completed treatment of 3 months at the standard recommended dosage of 4 capsules 3 times a day, and had data available on mRS scores at baseline and after 3 months of treatment. 

**Figure 1:**
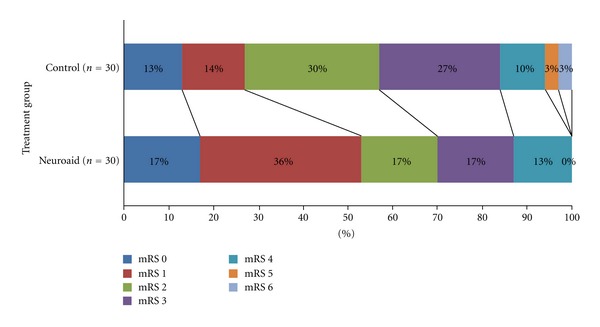
Distribution of mRS scores at 3 months.

In addition to age- and gender-matching, comparison stroke patients were consecutively identified from the same Stroke Data Bank if they met the same criteria above, but did not receive MLC601 yet had mRS assessments at the same time from stroke as the matching MLC601 patient (Figure 1).

All patients received standard stroke treatment as necessary and prescribed by the treating physician, including the use of antiplatelets, antihypertensives, hypoglycemic drugs, statins, and rehabilitation. 

The average number of day to initiation of treatment with the MLC601 regimen was 43 days from the time of stroke onset.

### 2.3. Statistical Analysis

Baseline characteristics collected were age, gender, medical history and vascular risk factors, prestroke and baseline mRS scores, and details of the index stroke including vascular distribution and the classification of the index stroke based on the Trial of Org 10172 in Acute Stroke Treatment (TOAST). The mRS scores at 3 months were obtained by reviewing the patient's outpatient records and via phone interview.

Baseline characteristics of the two groups were compared using Fisher's exact test for categorical variables and Student's *t*-test for quantitative data. The mRS scores at 3 months were analysed in three manners: by dichotomizing the mRS to either independent (mRS 0–2) or dependent (mRS 3–6), by dichotomizing mRS to either having returned back the prestroke mRS of 0-1 or not (mRS 2–6), and by comparison of mean mRS change from baseline to month 3.

The Mantel-Haenszel test was used to compare the proportion of patients who were independent 3 months after treatment and to compare the proportion of patients who achieved an outcome similar to prestroke mRS. Inverse variance method was used to assess the mean change of mRS score from baseline to 3 months. SPSS statistics version 17.0 (SPSS, Chicago, IL, USA) software was utilized for statistical analysis with a level of significance set at *P* < 0.05.

## 3. Results

Thirty MLC601-treated patients and 30 correspondingly matched non-MLC601 patients were identified using the criteria and were included in this analysis. Baseline characteristics including age, gender, mRS score at baseline, vascular distribution of strokes, classification based on the TOAST, and risk factors were similar between the two groups (Table 1).

The distributions of the mRS at 3 months for the MLC601 and non-MLC601 groups are shown in Figure 1. None of the MLC601 patients reported any serious adverse event during the 3-month course of treatment.

Among the MLC601-treated patients, 21 (70%) achieved functional independence defined as mRS 0–2 by the third month as compared to 17 (57%) in the non-MLC601 group although the difference did not reach statistical significance (OR 1.79; 95% CI 0.62–5.2; *P* = 0.29) (Table 2). However, there were twice as many patients who were able to achieve an mRS score of 0-1, which is similar to their prestroke conditions, in the MLC601 group (*n* = 16, 53%) as compared to the non-MLC601 group (*n* = 8, 27%) (OR 3.14; 95% CI 1.1–9.27; *P* = 0.038) (Table 2). While both groups showed statistically significant improvement in mRS scores from baseline to 3 months: by −1.7 (95% CI −1.35 to −1.98; *P* < 0.001) in the MLC601 group and −0.9 (95% CI −0.62 to −1.8; *P* < 0.001) in the non-MLC601 group (Table 3), the improvement was significantly better among the MLC601-treated patients with mean difference of −0.73, 95% CI −1.09 to −0.38; *P* < 0.001) (Table 4).

## 4. Discussion

In Asia, many poststroke patients seek alternative therapies due to dissatisfaction with their degree of recovery [17]. The utilization of traditional medicines has been part of stroke treatment in Asian countries such as in India and China [18]. Numerous articles in Chinese medical literatures regarding the usefulness and safety of traditional Chinese medicine (TCM) have been published [19]. However, most of these clinical trials have been of poor methodological quality [20].

MLC601 consists of 9 herbal (*Radix astragali*, *Radix salviae miltiorrhizae*, *Radix paeoniae rubra*, *Rhizoma chuanxiong*, *Radix angelicae sinensis*, *Carthamus tinctorius*, *Prunus persica*, *Radix polygalae,* and *Rhizoma acori tatarinowii*) and 5 animal (*Hirudo*, *Eupolyphaga seu steleophaga*, *Calculus bovisartifactus*, *Buthus martensii,* and *Cornu saigae tataricae*) components. Recent publications have shown benefit in the use of MLC601 in postischemic stroke patients. Many patients in these studies were nonacute and were included from within 1 week to up to 6 months since their stroke onset [8–15]. These studies offer an opportunity to intervene and improve functional and neurological outcomes further even if started in the recovery phase.

Our study looked at the same population of nonacute patients, but specifically using a well-established and most often used measurement tool for functional disability in stroke, the modified Rankin Scale [21]. We found that stroke patients given MLC601 in addition to standard treatment were more likely to attain better functional outcome without serious adverse effect after 3 months of treatment.

 We are very much aware of the limitations of this study. Outcome assessment bias may be reduced to a certain extent in our study since patients' information were systematically collected at the time they were included in the Stroke Data Bank, before the hypothesis being tested in this study was defined. However, it is difficult to avoid biases in an open-label, nonrandomized, nonblinded study, hence the large double-blind placebo-controlled randomized study will help confirm and validate the results observed in our small cohort.

 The exact mechanisms of MLC601 is yet unknown and MLC601 may very well act on many different pathways. However, the neuroprotective and, more importantly, neuroproliferative effects of MLC601 in animal models of focal and global ischemia [22, 23] is consistent with our clinical observation and that of other studies, and further strengthens the concept that intervention to reduce disabilities even long after the acute phase of a stroke may be feasible by enhancing neuroplasticity and neurogenesis. 

## Figures and Tables

**Table 1:** Baseline characteristics of MLC601-treated and -nontreated patients.

	MLC601 *N* = 30	Non-MLC601 *N* = 30	*P* value
Age, years	66 ± 11	65 ± 12	0.71
Female, *n* (%)	9 (30)	9 (30)	—
Baseline mRS score	3.4 ± 1.04	3.2 ± 1.3	0.083
Vascular distribution *n* (%)			
Left anterior circulation	14 (47)	15 (50)	0.99
Right anterior circulation	13 (43)	12 (40)	0.99
Posterior circulation	3 (10)	3 (10)	—
Classification of the Index Stroke Based on the TOAST Criteria *n* (%)			
Large artery atherosclerosis	18 (60)	19 (63)	0.99
Cardioembolism	5 (17)	4 (13)	0.99
Small vessel occlusion	7 (23)	7 (23)	—
Stroke of other determined etiology	0	0	—
Stroke of undetermined etiology	0	0	—
Risk factors *n* (%)			
Hypertension	25 (83)	27 (90)	0.70
Diabetes	9 (30)	9 (30)	—
Coronary artery disease	8 (27)	6 (20)	0.76
Dyslipidemia	18 (60)	18 (60)	—
Atrial fibrillation	5 (17)	4 (13)	0.99
Prior stroke	4 (13)	6 (20)	0.73
Average days to initiation of MLC601	44 days	—	—

**Table 2:** Results of statistical comparisons of mRS score at 3 months between MLC601 and non-MLC601 patients.

	MLC601 (*n* = 30)	Non-MLC601 (*n* = 30)	Odds ratio (95% CI)	*P* value
	*n* (%)	*n* (%)		

At 3 months				
mRS 0 to 2	21 (70%)	17 (57%)	1.79 (0.62 to 5.2)	0.29
mRS 0 to 1	16 (53%)	8 (27%)	3.14 (1.1 to 9.27)	0.038

**Table 3:** Mean difference (95% CI) in mRS score from baseline to 3 months.

	MLC601 (*n* = 30)	Non-MLC601 (*n* = 30)
Mean baseline mRS	3.4 ± 1.04	3.2 ± 1.3
Mean mRS at 3 months	1.7 ± 1.3	2.3 ± 1.5
Mean difference in mRS from baseline to 3 months (95% CI)	−1.7 (−1.35 to −1.98)	−0.9 (−0.62 to −1.8)
*P* value	<0.001	<0.001

**Table 4:** Comparison of the mean difference mRS between MC601 and non-MLC60 patients.

	MLC601 (*n* = 30)	Non-MLC601 (*n* = 30)	Odds ratio (95% CI)	*P* value
Mean difference in mRS between MC601 and Non-MLC601	−1.7 (−1.35 to −1.98)	−0.9 (−0.62 to −1.8)	−0.73 (−1.09 to −0.38)	<0.001
